# Heightened Circulating Interferon-Inducible Chemokines, and Activated Pro-Cytolytic Th1-Cell Phenotype Features Covid-19 Aggravation in the Second Week of Illness

**DOI:** 10.3389/fimmu.2020.580987

**Published:** 2020-10-20

**Authors:** Camilla Tincati, E. Stefania Cannizzo, Mauro Giacomelli, Raffaele Badolato, Antonella d’Arminio Monforte, Giulia Marchetti

**Affiliations:** ^1^Department of Health Sciences, Clinic of Infectious Diseases, ASST Santi Paolo e Carlo, University of Milan, Milan, Italy; ^2^Dipartimento di Scienze Cliniche e Sperimentali, Università degli Studi di Brescia, ASST Spedali Civili and A. Nocivelli Institute of Molecular Medicine, c/o Spedali Civili, Brescia, Italy

**Keywords:** Covid-19, SARS-CoV-2, immunity, S/M/N protein-reactive T-cells, immunopathology

## Abstract

Covid-19 features a delayed onset of critical illness occurring approximately one week from the beginning of symptoms, which corresponds to the bridging of innate and adaptive immunity. We reasoned that the immune events occurring at the turning point of disease might mark the direction toward pathogenic *versus* protective inflammatory responses. Subjects with either severe (s; PaO2/FiO2 ratio <200) or mild (m; PaO2/FiO2 ratio>300) Covid-19 were enrolled. A range of chemokines and cytokines as well as reactive oxygen species (ROS) were measured in plasma. Dendritic and NK cell frequency, monocyte and B-/T-cell phenotype and SARS-CoV-2-specific T-cell responses were assessed in PBMC. Twenty mCovid-19 and 20 sCovid-19 individuals were studied. sCovid-19 patients displayed higher non-classical monocytes, plasma chemokines (CXCL8, CXCL9, CXCL10), cytokines (IL-6, IL-10), and ROS *versus* mCovid-19. sCovid-19 also showed significantly increased activated CD38+HLA-DR+ T-lymphocyte, and granzyme-B+/perforin+ pro-cytolytic T-cells. All Covid-19 patients showed SARS-CoV-2 specific-T-cell response with a predominance of Th1 bi- or trifunctional IFN-*γ*/IL-2/TNF-*α*-expressing CD4+, while no difference according to disease severity was observed. Severe Covid-19 features heightened circulating IFN-inducible chemokines and activated pro-cytolytic Th1 cell phenotype in the second week of illness, yet SARS-CoV-2-specific responses are similar to that of mild illness. Altogether, our observations suggest Th1 polarization coupled to higher cytolytic profile in sCovid-19 as correlate of disease pathogenesis and as potential targets to be investigated in the roadmap to therapy and vaccine development.

## Introduction

SARS-CoV-2 is the etiologic agent of Coronavirus disease 2019 (Covid-19) which may feature interstitial pneumonia leading to severe respiratory distress and death (1). While literature findings point to the presence of older age, neutrophilia, organ, and coagulation dysfunction in subjects with critical disease (2–4), scant data exist on the immune correlates of Covid-19 progression.

Literature evidence suggests that individuals with severe SARS-CoV-2 infection may have a “cytokine storm syndrome”, characterized by increased levels of cytokines and chemokines (5–7), crucial mediators of the adaptive immune response.

Lymphopenia also appears to feature SARS-Cov-2 infection (1, 7, 8) with low CD4+, CD8+, B and NK counts (7, 9). In line with these findings, functional exhaustion of cytotoxic lymphocytes in adult individuals with Covid-19, presenting lower intracellular cytokine expression compared to healthy controls, has also been described (3). Of note, significantly lower T- and B-cell counts as well as skewing of T-cell maturation were detected in subjects with severe Covid-19 compared to those with mild disease (6, 9), suggesting that diverse adaptive immunity phenotypes may feature Covid-19 severity. In contrast, a limited number of studies report conflicting results on the functional profile of T-lymphocytes in SARS-CoV-2-infected subjects with different clinical presentation (6, 7).

Clinical aggravation of Covid-19 occurs approximately one week from the onset of illness (5, 10, 11), which corresponds to the temporal bridging of the innate and adaptive immune responses. In the animal model, CoV-specific T-cells are not only necessary and sufficient for virus clearance but also account for protection from clinical disease (12), as they dampen overactive innate immune responses (13, 14), thus limiting further damage to the host (12, 15). This observation has led us to hypothesize that the immune characteristics at this specific time-point in the course of SARS-CoV-2 infection may represent a watershed for the clinical outcome of the disease.

In the attempt to outline an immune signature of severe and mild Covid-19, we conducted an extensive analysis of innate and adaptive immune parameters of SARS-CoV-2-infected subjects at the defining moment of illness.

## Methods

### Study Population

We included in the study 40 individuals hospitalized with ascertained acute SARS-CoV-2 infection (positive naso-pharyngeal swab) presenting with either severe (n = 20, PaO2/FiO2 ratio< 200) or mild (n = 20, PaO2/FiO2 ratio>300) Covid-19. At hospitalization, demographic, and clinical characteristics were recorded from electronic clinical charts. All enrolled patients provided written informed consent according to the Ethical Committee of our institution (no. 2020/ST/049). Following informed consent, peripheral blood samples were collected from all study participants for plasma and peripheral blood mononuclear cell (PBMC) separation which were stored for laboratory analyses.

Immune parameters were compared to those of 10 healthy controls (HC) in archived laboratory samples.

### Plasma Chemokine and Cytokine Measurements

Chemokine (CXCL8/IL-8, CCL5/RANTES, CXCL9/MIG, CCL2/MCP-1 and CXCL10/IP-10) and cytokine (IFN-*α*, IFN-*γ*, IL-1β, IL-4, IL-5, IL-6, IL-10, IL-12 (p70), IL-17A, and tumor necrosis factor-α) serum levels were analyzed with Bead Array Human Chemokine Kit, and Bead Array Human cytokine Kit (Becton Dickinson, San Josè, CA) following the manufacturer’s instructions.

Briefly, the samples were diluted 1 to 4 with assay diluent and incubated for 3 h with the relative capture beads and with the human chemokine detection reagent. Samples were washed in a wash buffer resuspended in 0.3 ml of the same buffer and acquired by flow cytometry. Data were acquired on (BD) FACSCanto II flow-cytometer and analyzed by FCAP v3 array software.

### Cell Phenotyping

Lymphocyte surface phenotypes were evaluated by flow cytometry on cryopreserved PBMCs: CD4-APC-H7, CD8-PErCP-Cy5.5, CD38-PCY-7, HLA-DR-BV421, CD45RA-APC-H7, CCR7- PECy5, LIVE/DEAD-V500, Granzyme B-PE, Perforin-FITC, CD16-APC, CD14-BV421, CD56-PE (BD Biosciences, San Jose, CA, USA) and CD19-PercPVio700, CD80-APC (Miltenyi Biotec, Bergisch Gladbach, Germany). Combinations used were: CD4/CD8/CD38/HLA-DR (T-cell activation), CD14/CD16 (monocyte), CD16/CD56/CD3 (NK cells), CD11c/CD3 (DC), CD3/CD19/CD80 (B-cell activation), CD4/CD8/CD45RA/CCR7 (T-cell maturation). T-cell subsets were defined as naïve CCR (C-C chemokine receptor)7+CD45RA+, central memory (CM) CCR7+CD45RA−, effector memory (EM) CCR7−CD45RA−, terminally differentiated (TD) CCR7−CD45RA+. T follicular helper (Tfh) CD4+CxCR5+CD45RA+, T helper 17-like (Th17) CD4+CCR6+CD161+, T regulatory-like (Treg) CD4+CD127-CD25+. Briefly, 1 × 10^6^ PBMCs were stained with the appropriate antibodies for 20 min at 4°C in the dark and then washed and acquired using FACSVerse™ cytometer (BD Biosciences).

### Antigen Stimulation

SARS-CoV-2 PepTivator peptide pools (Miltenyi Biotec, Bergisch Gladbach, Germany), consisting of the S-protein, N- and M-protein pools, were used for PBMC stimulation, given data on M, spike, N co-dominance in T-cell SARS-CoV-2-specific response (16). PBMCs were prepared from EDTA collection tubes by gradient centrifugation. 1.5 × 10^6^ PBMCs were stimulated with 1 μg/ml peptide pool for 16 h in RPMI supplemented with 1% Penicillin–Streptomycin–Glutamin and 10% FCS or Phorbol myristate acetate (PMA) (25 ng/ml) and ionomycin (Sigma Aldrich Merck) (1 ug/ml). 1.5 ×10 ^6^ PBMCs were also stimulated with *Staphylococcus aureus* enterotoxin B (SEB, Sigma-Aldrich, Darmstadt, Germany) at 1 μg/ml as a positive control (**Supplementary Figure 2**). Negative controls were left untreated. Brefeldin A (1 μg/ml, Sigma Aldrich) was added after 1 h. Intracellular detection of IL-17A, IL-4, IL-2, interferon (IFN)-*γ* and tumor necrosis factor (TNF)-α was assessed by flow cytometry. Only samples with >90% cell vitality were studied. Antibodies used were: CD4-APC-H7, CD8-PECy5, LIVE/DEAD-V500, IL-17A-FITC, IL-4-PE, TNF-A-V450, IFN-Y-PECY-7, IL-2-APC (BD Biosciences, San Jose, CA, USA) (Miltenyi Biotec, Bergisch Gladbach, Germany). Cells were harvested after stimulation and stained with surface antibodies; after paraformaldehyde (PFA) fixation (1%, Sigma-Aldrich), cells were permeabilized with Saponin 0.2% (Sigma-Aldrich) and stained with intracellular cytokines for 30 min at room temperature (RT). Unspecific activation in unstimulated controls was subtracted from stimulated samples to account for specific activation. We considered a positive response when the cytokine production was above the 90^th^ percentile of healthy controls.

### Soluble ROS Quantification

Plasma levels of ROS were measured by an enzyme-linked immunosorbent assay (ELISA; LSBio), according to the manufacturer’s instructions.

### Statistics

Twenty individuals with severe and 20 with mild Covid-19 (sCovid-19 and mCovid-19, respectively) were enrolled. Aside for a non-significant trend to older age in sCovid patients, no significant differences were registered between groups in terms of sex, comorbidities, type, and duration of Covid-19-related symptoms (**Table 1**). The two groups were comparable in terms of pulmonary radiologic findings and medical therapy, except for a higher proportion of subjects with sCovid-19 on supplemental oxygen upon hospital admittance and more frequent treatment with Continuous Positive Airway Pressure (CPAP) and mechanical ventilation during hospitalization (**Table 1**).

**Table 1:** Demographic and clinical characteristics of study subjects.

Characteristic	All Covid-19 N = 40	Severe (s) Covid-19 N = 20	Mild (m) Covid-19 N = 20	p-value sCovid-19 *vs* mCovid-19
**Sex (n, %)°**				1.0
M	33 (83)	17 (85)	16 (80)	
F	7 (17)	3 (15)	4 (20)	
Age, years (median, IQR)*	61, 53–72	64, 55–77	56, 47–63	0.06
**PaO2/FiO2**	206, 88–341	86, 69–141	340, 304–391	<0.0001
**Symptoms upon hospitalization (n, %)°**				
Fever Cough Dyspnea Thoracic pain Conjunctivitis Diarrhea	37 (93) 26 (65) 19 (48) 5 (13) 1 (3) 2 (5)	18 (90) 12 (60) 11 (55) 2 (10) 0 (0) 0 (0)	19 (95) 14 (70) 8 (40) 3 (15) 1 (5) 2 (10)	0.5 0.7 0.5 1.0 1.0 0.5
Duration of symptoms, days (median, IQR)*	7, 3–8	7, 5–8	6, 2–8	0.3
**Comorbidities (n, %)°**				
Hypertension Diabetes Cardiovascular Disease Pulmonary Disease Renal Disease Neoplasms	13 (33) 4 (10) 5 (13) 6 (15) 2 (5) 4 (10)	6 (30) 3 (15) 2 (10) 5 (25) 1 (5) 3 (15)	7 (35) 1 (5) 3 (15) 1 (5) 1 (5) 1 (5)	1.0 0.6 1.0 0.2 1.0 1.0
White Blood Cells, cell/µl (median, IQR)*				
Neutrophils Lymphocytes Monocytes	6,190, 4,580–8,393 4,360, 2,778–6,513 970, 720–1,228 470, 350–560	7,860, 5,458–9,413 6,040, 4,130–8,373 800, 635–1,040 445, 285–545	4,880, 4,390–6,608 3,375, 2,670–4,365 1,170, 963–1,538 530, 390–650	0.02 0.004 0.004 0.2
Platelet Count, cell ×10^3^/µl (median, IQR)*	232, 184–289	253, 132–293	230, 197–270	0.9
Inflammation Markers (median, IQR)*				
C Reactive Protein, mg/L Ferritin, ng/ml	56, 19–111 542, 209–818	109, 65–129 549, 346–849	33, 15–48 172, 117–912	<0.0001 0.4
LDH, U/L (median, IQR)*	295, 228–379	364, 284–566	237, 204–301	0.0004
Coagulation (median, IQR)*				
INR D-dimer, ng/ml	1, 1–1.2 394, 305–835	1, 1–1 414, 367–1,514	1, 1–1 202, 135–550	0.5 0.01
Liver enzymes, U/L (median, IQR)*				
AST ALT	33, 29–57 38, 24–49	52, 31–80 39, 26–84	30, 26–33 30, 22–42	0.001 0.2
Creatinine, mg/dl (median, IQR)*	0.8, 0–1	0.7, 0–1	0.85, 0.13-1.0	1.0
Total Bilirubin, mg/dl (median, IQR)*	0.4, 0–1.1	0.2, 0–1	0.5, 0–1	0.6
**Pulmonary infiltrates**				
None Monolateral Bilateral	2 (5) 10 (25) 28 (70)	0 (0) 4 (20) 16 (80)	2 (10) 6 (30) 12 (60)	0.5 0.7 0.3
Medical therapy*				
LPV/r or DRV/c Hydrossicloroquine Antibiotics Steroids	30 (75) 33 (83) 18 (45) 3 (8)	17 (85) 19 (95) 11 (55) 3 (15)	13 (65) 14 (70) 7 (35) 0 (0)	0.3 0.1 0.3 0.2
Oxygen therapy upon admittance (n, %)*				
None/nasal cannula Mask/Reservoir CPAP	27 (68) 9 (23) 4 (10)	8 (40) 8 (40) 4 (20)	19 (95) 1 (5) 0 (0)	0.0004 0.02 0.1
Oxygen therapy during hospitalization (n, %)*				
None/nasal cannula Mask/Reservoir CPAP	18 (45) 2 (5) 18 (45)	0 (0) 2 (10) 16 (80)	18 (90) 0 (0) 2 (10)	<0.0001 0.5 <0.0001
**Mechanical Ventilation**				
Non-Invasive Invasive	8 (20) 7 (18)	8 (40) 7 (35)	0 (0) 0 (0)	0.003 0.008
**Outcome**				<0.0001
Death Dismissal	15 (37) 25 (63)	14 (70) 6 (30)	1 (5) 19 (95)	

*Data are median (IQR). IQR, Interquartile range, Statistical analyses: Mann–Whitney U Test. Data are n (%), Statistical analyses: Pearson Chi squared or Fisher Exact Test; p values are referred to the comparison between the two study groups where applicable.

Furthermore, sCovid-19 patients presented significantly higher CRP, d-Dimer and LDH levels, total white blood cells and neutrophils *versus* mCovid-19 (**Table 1**). Death as a clinical outcome was more frequent in sCovid-19 than in mCovid-19 (**Table 1**).

Samples for immune investigations were collected at a median (IQR) of 7 (3–8) days from symptoms onset, with no differences between mCovid and sCovid patients (**Table 1**).

### Innate Immune Cells

**Figure 1:**
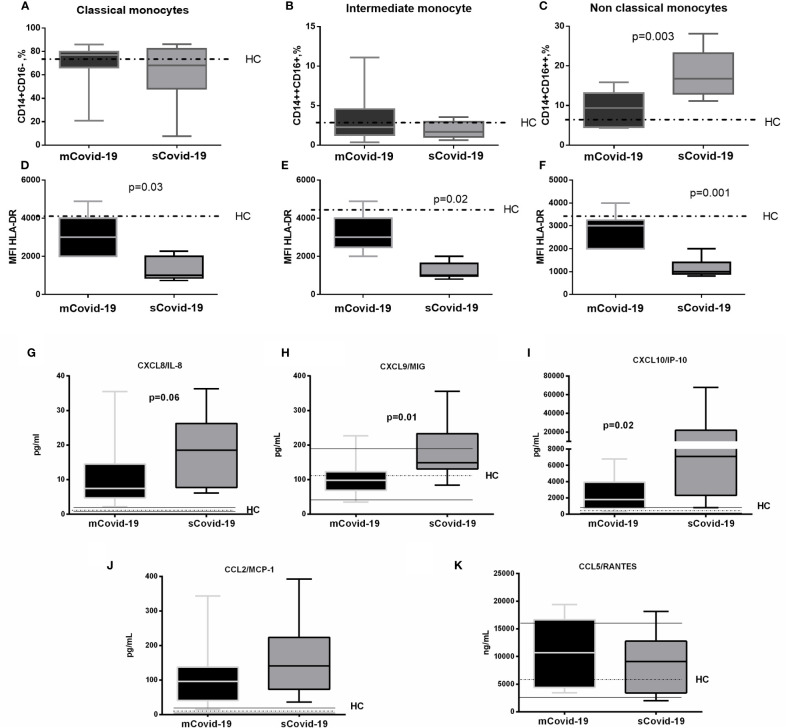
Frequency and phenotypes of monocyte subsets and levels of plasma chemokines according to the degree of Covid-19 diseases. Peripheral blood mononuclear cells (PBMCs) were isolated and analyzed from blood samples of 40 COVID-19 patients [severe Covid-19 (sCovid-19) n = 20 and mild Covid-19 (mCovid-19) n = 20]. **(A, B)** No differences were observed in classical monocytes (CD14+CD16−) and in intermediate monocytes (CD14++CD16+) between the two study groups. **(C)** Non-classical monocytes (CD14+CD16++) were increased in (sCovid-19) compared to (mCovid-19). **(D–F)** HLA-DR mean fluorescent intensity (MFI) on monocytes is lower in sCovid compared to mCovid patients. **(G)** CXCL8/IL-8 plasma levels were increased in sCOVID-19 patients in comparison to mCOVID-19 patients, albeit not reaching significance (p  =  .06). **(H, I)** sCOVID-19 patients showed significantly higher circulating levels of CXCL9/MIG and CXCL10/IP-10 in comparison to mCOVID-19 patients (p  =  0.01 and p = 0.02) **(J, K)** When mCOVID-19 patients were compared to sCOVID-19 patients, no differences in CCL5/RANTES and CCL2/MCP-1 plasma levels were detected. Graph box and whiskers represent medians and 10^th^**–**90^th^ range percentile range. Dotted lines indicate the median levels, and solid lines indicate 10^th^**–**90^th^ range of healthy controls (HC) from archived material. Comparison between groups (sCovid-19 *vs* mCovid-19) Mann**–**Whitney test.

Comparable frequencies of dendritic cells (DC, CD11c+) and natural killer cells (NK, CD56+CD16+) were observed between groups (**Supplementary Table 1**), as well as classical (CD14+CD16−) and intermediate monocytes (CD14++CD16+) (**Figures 1A, B**). In sharp contrast, sCovid-19 patients presented significantly higher percentages of non-classical monocytes (CD14+CD16++) *versus* mCovid-19 (16.8 *vs* 9.8%, p = 0.003 **Figure 1C**). Interestingly, compared to mCovid-19, sCovid-19 displayed decreased HLA-DR molecules on all three monocyte subtypes (**Figures 1D–F**). Whereas no differences between groups were detected in CXCR5 and CD45RA monocyte expression (**Supplementary Table 1**).

### Plasma Chemokines, Cytokines and ROS Levels

**Figure 2:**
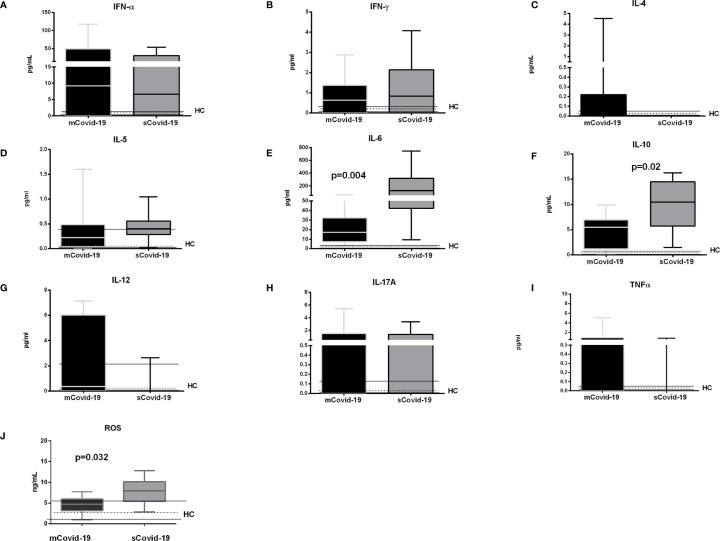
Levels of plasma cytokines and oxidative stress according to the degree of Covid-19 diseases. Plasma samples were collected and analyzed from blood samples of 20 COVID-19 patients [severe Covid-19 (sCovid-19) n = 10 and mild Covid-19 (mCovid-19) n = 10]. **(A–D)** No major differences in Interferon (IFN)-α, IFN-*γ*, Interleukin (IL)-4 and IL-5 were observed between (mCovid-19) and (sCovid-19). **(E, F)** We observed increased levels of IL-6 and IL-10 in sCovid-19 compared to m-Covid-19 (p = 0.004 and p = 0.02). **(G–I)**. The levels of IL-12, IL-17A and tumor necrosis factor (TNF)-α were comparable between mCovid-19 and sCovid-19. **(J)** sCOVID-19 patients showed significantly higher circulating levels of reactive oxygen species (ROS) in comparison to mCOVID-19 patients (p  =  0.032). Graph box and whiskers represent medians and 10^th^–90^th^ percentile range. Dotted lines indicate the median levels, and solid lines indicate 10^th^–90^th^ range of healthy controls (HCs) from archived material. Comparison between groups (sCovid-19 *vs* mCovid-19) Mann–Whitney test.

Compared to mCovid-19, sCovid-19 subjects presented a trend to higher circulating CXCL8/IL-8 (8 pg/ml, 5**–**15 *vs* 19 pg/ml, 8**–**26; p = 0.06; **Figure 1G**), higher CXCL9/MIG (98 pg/ml, 69**–**123 *vs* 149 pg/ml, 131**–**233; p = 0.01; **Figure 1H****)**, and CXCL10/IP-10 (1,808 pg/ml, 729**–**3949 *vs* 7,069 pg/ml, 2,299**–**21,893; p = 0.02; **Figure 1I**). Likewise, sCovid-19 presented higher CCL2/MCP-1, albeit not reaching statistical significance (96 pg/ml, 41**–**137 *vs* 141 pg/ml, 73**–**223; p = 0.22; **Figure 1J**), whereas no differences between groups were detected in CCL5/RANTES (10,680 ng/ml, 4,424**–**16,645 *vs* 9,094 ng/ml, 3,413**–**12,774; p = 0.54; **Figure 1K**). Interestingly, the frequency of circulating neutrophil cells was positively associated with CXCL9/MIG, CXCL10/IP-10 and CXCL8/IL-8 (*r* = 0.35, *p* = 0.05; *r* = 0.39, *p* = 0.05 and r = 0.5, p = 0.01). No major differences in Interferon (IFN)-a, IFN-g, Interleukin (IL)-4 and IL-5 were observed between (mCovid-19) and (sCovid-19) (**Figure 2A–D**).

Covid-19 patients also displayed greater IL-6 levels (17 pg/ml, 7**–**32 *vs* 126 pg/ml, 42**–**318; p = 0.004; **Figure 2E**) and IL-10 (5 pg/ml, 1**–**7 *vs* 11 pg/ml, 6**–**15; p = 0.02; **Figure 2F**). IL-6 values positively correlated with both neutrophils (r = 0.6, p = 0.004) and classical and non-classical monocytes (r = 0.6, p = 0.0008, and r = 0.5, p = 0.006). The levels of IL-12, IL-17A and tumor necrosis factor (TNF)-a were comparable between mCovid-19 and sCovid-19 (**Figure 2G–I**).

Further, reactive oxygen species (ROS), which derive, among others, from non-classical monocytes and neutrophils, were also greater in sCovid-19 subjects (7.9 *vs* 4.7 ng/ml; p = 0.032) (**Figure 2J**), and correlated with the frequencies of both non-classical monocyte (r = 0.44, p = 0.042); and neutrophil frequencies (r = 0.40, p = 0.01).

### T-Cell Immunephenotype

**Figure 3:**
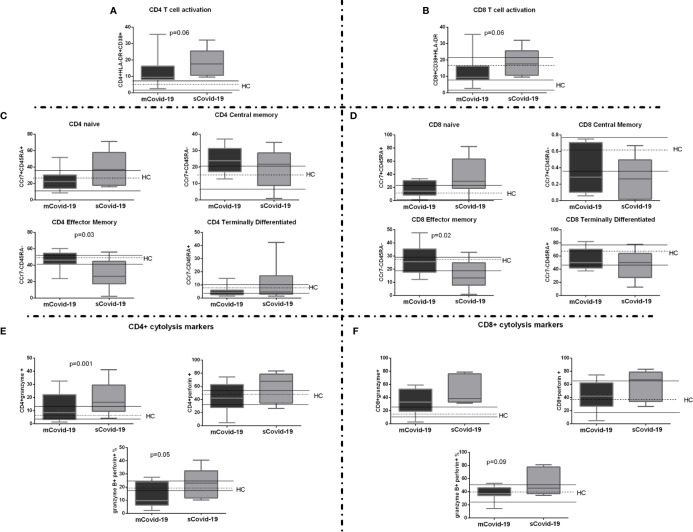
T-cell activation, maturation, and cytolysis markers among COVID-19 patients. Peripheral blood mononuclear cells (PBMCs) were isolated and analyzed from blood samples of 40 COVID-19 patients [severe Covid-19 (sCovid-19) n = 20 and mild Covid-19 (mCovid-19) n = 20]. **(A, B)** HLA-DR+CD38+ expressing both in CD4+ and CD8 T-cells were significantly increase in (sCovid-19) compared to (mCovid-19) (p = 0.06 and p = 0.06). **(C**, **D)** We observed no difference in naïve (CCR7+CD45RA+), CM: central memory (CCR7+CD45RA−) and TD: terminally differentiated (CCR7−CD45RA+). sCOVID-19 patients showed significantly lower CD4+ and CD8+ EM: effector memory (CCR7−CD45RA−) (p = 0.03 and p = 0.02). **(E)** Compared to mCovid-19, sCovid-19 patients tended to have a higher number of CD4+granzyme+ cells (p = 0.001). We fail to observe any modification in CD4+ perforin + cells. The number of CD4+granzyme+perforin+ cells was higher in sCovid-19 patients when compared to mCovid-19 (p = 0.05). **(F)** No major differences in CD8+ granzyme+ and CD8+ perforin+ were observed between mCovid-19 and sCovid-19. A non-significant tendency towards increased CD8+ granzyme-perforin+ frequency was observed in sCovid-19 patients in comparison to mCovid-19 (p  =  .09). In each graph the columns represent the median values, while the error bars indicate the 10^th^–90^th^ percentile range. Dotted lines indicate the median levels and solid lines indicate 10^th^–90^th^ percentile range of healthy controls (HCs) from archived material. Comparison between groups (sCovid-19 *vs* mCovid-19) Mann–Whitney test.

Circulating total CD3+, CD4+ and CD8+ T-lymphocytes were comparable in mCovid and sCovid patients (**Supplementary Table 1**). Interestingly, sCovid displayed a trend to significantly higher activated HLA-DR+CD38+CD4+ and CD8+ *versus* mCovid (p = 0.06 for both comparisons; **Figures 3A, B**).

We next investigated T-cell maturation, finding significant differences according to disease severity, with sCovid-19 patients displaying lower EM cells (p = 0.03; **Figure 3C** and p = 0.02; **Figure 3D**) and higher granzyme B- and perforin-producing CD4+ (**Figure 3E**). A similar trend was observed in CD8+ compartment, albeit not reaching statistical significance (**Figure 3F**).

sCovid-19 and mCovid-19 displayed comparable Treg, Th17 and TFh (**Supplementary Table 1**).

### T-Cell Cytokine Secretion Upon SARS-CoV-2 Challenge in Covid-19 Patients

Because CoV-specific T-cells are crucial for virus clearance, we sought to investigate *ex vivo* CD4+/CD8+ T-cell intracellular cytokine production following *in vitro* challenge by PMA (positive control), SARS-CoV-2 peptide pool (gating strategy in **Supplementary Figure 1**) and SEB (**Supplementary Figure 2**). It must be noted that although IL-4 and IFN-*γ* were positively produced following PMA stimulation, the frequency of CD4^+^ T-cells producing IL-4 and IFN-*γ* were lower than expected.

**Figure 4:**
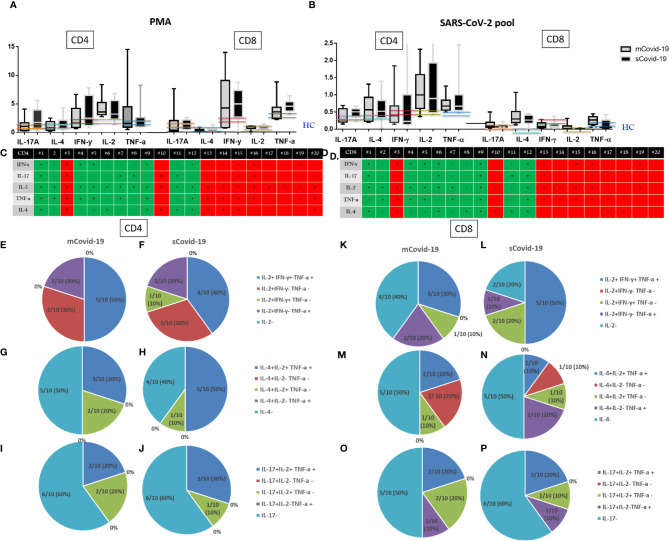
T-cell cytokine secretion upon phorbol myristate acetate (PMA)/ionomycin, and Sars-Cov-2 challenge: Severe Covid-19 (sCovid-19) *vs* mild Covid-19 (mCovid). Peripheral blood mononuclear cells (PBMCs) were isolated from blood samples of unselected 20 COVID-19 patients (n = 10 sCovid-19 and n = 10 mCovid-19). PBMCs were incubated for 16 h with SARS-CoV-2 spike (S)-, membrane (M)-, and nucleocapsid (N)-protein peptide pools and analyzed with flow cytometry. **(A**, **B)** Frequencies of interferon-*γ* (IFN-*γ*)−, tumor necrosis factor-α (TNF-α)−, interleukin (IL)-2−, IL-4−, and IL-17A producing antigen-specific CD4+ and CD8+ T-cells upon **(A)** Phorbol myristate acetate (PMA) and ionomycin, **(B)** Sars-Cov-2 pool. In each graph, the columns represent the median values, while the error bars indicate the 10^th^–90^th^ percentile range. Frequencies were corrected by background subtraction as determined in non-stimulated controls. Dotted lines indicate the median levels of healthy controls (HC) from archived material. No major differences in CD4 and CD8 cytokine productions were observed between mCovid-19 and sCovid-19. Comparison between groups (sCovid-19 *vs* mCovid-19) Mann–Whitney test. **(C**, **D)** Tables represent the total production of different cytokines for all enrolled patients. Green boxes represent mCovid-19 patients, and red boxes represent sCovid-19 patients. **(E**–**P)** Composition of bi- and trifunctional CD4+ and CD8+ T-cells after stimulation with Sars-Cov-2 pool. The fraction of positive subsets (above 90^th^ percentile of healthy controls) of all bi- or trifunctional cells was calculated for each combination of cytokine. Colored lines indicate 90^th^ and 10^th^ percentile ranges of healthy controls.

Although we detected both CD4+ and CD8+ SARS-CoV-2-specific T-cell responses, the frequency and magnitude of CD4+ response was greater than CD8+ (**Figure 4**).

Covid-19 subjects as a whole presented higher CD4+ and CD8+ IL-17A, IL-4, IFN-*γ*, IL-2, TNF-α cytokine production in response to SARS-CoV-2-specific stimulation compared to HC from archived materials who were not exposed to SARS-CoV-2 infection (**Figure 4**). This suggests a specific T-cell response in Covid-19 patients, while we could not detect differences in intracellular cytokine production between mCovid-19 and sCovid-19 subjects (**Figure 4**).

Because we found that SARS-CoV-2-reactive CD4+ T-cells were predominantly of Th1 phenotype, we next analyzed polyfunctionality by IFN-*γ*, TNF-α, IL-2, IL-4, IL-17 expression in parallel.

Following SARS-CoV-2 antigens challenge, 20/20 patients presented IL-2-producing CD4+, of which 45% (9/20) displayed trifunctional IL-2+IFN-*γ*+TNF-α+CD4+, 6/20 (30%) single IL-2+ IFN-*γ*−TNF-α−CD4+, 5/20 (25%) bifunctional CD4+ (1/20, 5% IL-2+IFN-*γ*+TNF-α−, and 4/20, 20% IL-2+IFN-*γ*−TNF-α+). Dissecting CD8+ Th1 intracellular cytokine expression, 30% (6/20) of patients lacked IL-2-expressing CD8+, 8/20 (40%) patients displayed a trifunctional IFN-*γ*+IL-2+TNF-α+CD8+ subset, while 6/20 (30%) showed bifunctional CD8+ (3/20, 15% IL-2+IFN-*γ*+TNF-α−, 3/20, 15% IL-2+IFN-*γ*−TNFα+).

Intracellular IL-4 cytokine staining revealed IL-4 producing CD4+ in 11/20 (55%), of which 8/11 (73%) were trifunctional IL-4+IL2+TNFα+, 3/11 (27%) were bifunctional (IL-4+IL-2+TNF-α−).

Likewise, 10/20 (50%) patients presented IL-4-producing CD8+, of which 30% (3/10) show trifunctional IL-4+IL2+TNF-α+, 40% (4/10) bifunctional (2/20, 10% IL-4+IL-2+TNF-α−, and 2/10, 20% IL-4+IL-2−TNF-α+), and 30% (3/10) single IL-4-expressing CD8+ phenotype.

We lastly investigated IL-17-producing phenotypes. Overall, 8/20 (40%) patients presented IL-17-producing CD4+ T-cells, of which 5/8 (62%) were trifunctional IL-17+IL-2+TNF-α+ and 3/8 (37%) bifunctional IL-17+IL-2+TNFα−CD4+.

Likewise, 9/20 (45%) patients present IL-17-producing CD8+: 4/9 (44%) trifunctional IL-17+IL-2+TNF-a+, and 5/9 (55%) bifunctional CD8+ (3/9, 33% IL-17+IL-2+TNF-α− and 2/9, 22% IL-17+IL-2−TNF-a+).

Interestingly, breaking down CD4+/CD8+ SARS-CoV-2-specific Th1, Th2, and Th17 intracellular cytokine data, no differences were shown in the proportion of polyfunctional CD4+/CD8+ subsets in mCOVID-19 *versus* sCOVID-19 (**Figures 4E–P**).

## Discussion

Covid-19 pandemic has dramatically struck Italy: as of September 7th, Italy recorded 277,634 laboratory-confirmed cases (>35,000 deaths) with over 100,000 in Lombardy (17, 18).

Although about 80% of Covid-19 patients display a benign clinical phenotype, up to 20% of the patients can develop rapidly progressing respiratory failure. While several clinical and epidemiological factors have been associated with poor outcome, specific immunologic aspects featuring the worst clinical outcome are still elusive. Clinical experience with Covid-19 has demonstrated that the second week of illness seemingly represents a turning point in disease, suggesting that the immune events occurring at this phase of the infection might mark the direction toward pathogenic *versus* protective inflammatory responses.

With this idea in mind we comparatively assessed innate and adaptive immunity measured at approximately one week from the onset of symptoms in a cohort of Covid-19 patients featuring severe *versus* milder illness.

In patients developing severe Covid-19, we demonstrate: i) elevated inflammatory chemokines, cytokines and ROS positively associating with neutrophilia and pro-inflammatory monocytes; ii) T-cell immune phenotype characterized by increased activated, granzyme/perforin-producing T-lymphocytes, and reduced effector-memory cells; iii) evidence of SARS-CoV-2-specific intracellular cytokine production, with a predominance of Th1 CD4+ T-cells, similar to patients with milder disease.

The direct comparison of patients with severe *versus* moderate disease revealed highest inflammatory chemokines including MCP-1/CCL2, IP-10/CXCL10, IL-8/CXCL8, as well as cytokines in subjects with critical illness, correlating with both neutrophilia and increased circulating pro-inflammatory CD14+CD16++ monocytes with reduced HLA-DR surface expression.

In particular, while finding elevated circulating IL-6 and decreased HLA-DR expression on circulating monocytes in sCovid-19, we describe less relevant changes in other pro-inflammatory cytokines, in line with the predominant role of IL-6 as driver of Covid-19 hyperinflammatory response, immune dysregulation and respiratory failure (19–21).

The behavior of other pro-inflammatory cytokines in Covid-19 has proven more erratic across the literature, with discordant findings being published (6, 22, 23). In particular, the failure to detect differences in IL-1β and TNF-α might reflect receptor antagonism or differential cytokine concentrations in diseased tissue and peripheral blood (24).

Having ascertained a pattern of “cytokine storm” (6, 22, 25–27), more evident in patients with severe disease, and given the crucial interplay between innate and adaptive immunity, we next investigated T-cell responses, aiming to shed light on the contribution of adaptive immunity on Covid-19 course.

To date, little is known about the antiviral T-cell responses in Covid-19. In animal models of coronavirus infection, adaptive T-cell immunity has proven essential in tempering the innate immune response, in turn mitigating immunopathology. Indeed, upon acute coronavirus infection, T-cell deficient mice mounted an exaggerated innate immune response with high levels of circulating pro-inflammatory cytokines, resulting in rapid lethality, proving that an unleashed innate response together with the lack of antiviral-specific responses can be a direct cause of death (12, 13, 28).

Less is known about T-cell response in human coronavirus, whether it contributes to disease progression or recovery (25, 29). In MERS, SARS, and Covid-19, patients with severe/fatal outcomes present progressive lymphopenia and neutrophilia peaking at approximately days 7–10 from symptom onset, while healing patients efficiently recover physiologic lymphocyte/neutrophil counts, suggesting a crossover between innate and adaptive immunity in dictating disease outcome, with adaptive immunity on the one hand controlling the infection and on the other hand slowing immune pathology (6).

Accordingly, in our patient cohort, despite similar demographic and pre-existing comorbidities, as well as analogous lung damage, we show reduced neutrophil and increased lymphocyte counts in moderately *versus* severely ill individuals at an average of 7 days from symptom onset. Because lymphocyte activation/exhaustion has been suggested in Covid-19 adult patients (3, 22), we next characterized T-cell immunophenotype and function according to disease severity, with particular focus on SARS-Cov-2-specific response.

As compared to milder disease, sCovid patients show highly activated CD4+/CD8+ T-cells, with reduced effector-memory and raised pro-cytolytic phenotypes. Given the paradigm of T-cell differentiation described in humans featuring naïve→central-memory→effector-memory→terminally-differentiated (30), our findings of higher proportion of activated T-cells, with lower effector-memory cells and higher cytolytic potential in s- *versus* mCovid patients would altogether suggest higher T-lymphocyte activation upon acute SARS-CoV-2 infection in individuals with a severe disease course, resulting in the continuous T-cell differentiation, overall resulting in a net reduction of the effector-memory at the advantage of cytolytic T-cell phenotype. Having shown that despite their bad prognosis, sCovid patients display more vigorous T-lymphocyte engagement, activation and function, we next sought to investigate SARS-CoV-2-specific response according to disease severity.

Interestingly, we demonstrate the presence of T-cell reactivity to SARS-CoV-2 S-, M-, N- overlapping antigen pool, at higher magnitude within the CD4+ compartment, confirming the pivotal role of CD4+ in the control over SARS-CoV infection, confirming data on co-dominant M, spike and N-specific CD4+ response in 100% of Covid-19 convalescent patients (16), as well as both humans and animal data correlating disease severity and CD4+ responses in the course of SARS (25, 31).

Further detailing T-cell response revealed virus-specific IL-2-producing CD4+ in the whole Covid-19 cohort, with up to 75% of the patients displaying bi- or trifunctional INF-*γ/*IL-2/TNF-α-expressing CD4+ and similar virus-specific CD8+ functionality despite lower frequency and magnitude. Because polyfunctional T-cell response has been associated with better immunity *versus* several infections (32–34), our data indicate an ongoing Th1-polarized response to SARS-Cov-2, in agreement with recent data showing predominant Th1 responses in convalescent (16) as well as in ARDS ICU patient cohort (20).

Unexpectedly however, despite a more activated/pro-cytolytic T-lymphocyte asset, we failed to detect any difference in virus-specific intracellular cytokine response according to disease severity. In particular, despite data suggesting Th2 polarization as correlate of immunopathology (25), about half of our patients presented IL-4-expressing CD4+ irrespective of disease outcome, with a reasonable proportion of IL-4 and Th1 cytokine-co-expressing T-cells to possibly suggest a Th0 profile in this stage of the disease. Given recent data showing a predominant Th1 response in a small but well-defined cohort of convalescent uncomplicated non-hospitalized Covid-19 cases (16), it will be interesting to longitudinally follow up the fate of Th1/Th2 ratio in later disease phases as well as in patients recovering from complicated *versus* uncomplicated disease.

Likewise, our finding of a relevant proportion of IL-2/TNF-α/IL-17 co-expressing T-cells suggest the activation of IL-17-mediated immune pathway whose role in neutrophil recruitment and immune regulation in Covid-19 will need to be further investigated.

Collectively, our data provide some hints to better understand Covid-19 pathogenesis. Together, the findings of similar plasma IFN-*γ*, higher circulating IFN-stimulated chemokines CXCL9 and CXCL10 (35) in sCovid, and an overall heightened Th1 virus-specific *ex vivo* T-cell response, suggest Th1 polarization. The lack of difference in IFN-*γ* plasmatic levels might reflect a more vigorous response in lymphoid organs and diseased tissues (24) that might therefore fail to be captured by peripheral blood cytokine assessment, in accordance with *post-mortem* data showing mononuclear cell accumulation in the lungs (36).

Interesting speculations derive from the investigation of T-lymphocyte phenotype and function. While the prevalent activated/cytolytic T-cell phenotype would indicate vigorous T-cell activation and function to neutralize the infection, the lack of a difference in virus-specific T-cell response in sCovid *versus* mCovid was somehow unexpected and contrasts previous data in SARS (25). Higher T-cell activation and differentiation in the face of non-efficacious virus-specific response have been described in other models of viral infections such as HIV (37, 38), where ongoing non-virus-specific immune activation has been long proven a major driver of disease progression even after viral abatement by antiretroviral therapy (39, 40).

As a caveat in the interpretation, it must be noted that all but two Covid-19 patients in our cohort developed pneumonia: it will be interesting to assess SARS-Cov-2 specific T-cell responses in pauci-symptomatic patients without pneumonia. However, 14/15 patients who died were within the sCovid groups, so we can assume that the immune picture that we describe realistically captures the immune events contributing to the most severe immune pathology and clinical prognosis.

Limitations of this study include the patient’s size and the lack of a longitudinal assessment that was not possible given the high mortality within the sCovid group. In analogy to what was described in other models of infectious diseases where disease progression has been associated with different profiles of virus-specific T-cell functionality (34, 41), a detailed longitudinal profiling of virus-specific intracellular cytokine asset in larger patients cohorts with different disease phenotype will further inform on the immune features and tempo of disease progression and severity.

While the hectic run to anti-SARS-Cov2 therapy and vaccine is ongoing, our study adds to the body of literature aimed at broadening the knowledge about T-cell responses (42).

While no current targeted treatment is available thus far, combined antiviral, antimalarials, corticosteroids, anti-inflammatory molecules, convalescent plasma, and anticoagulant approaches are being used and investigated for the treatment of Covid-19 (43). Among immunomodulants, biologics interfering with the cytokine storm, mainly the IL-6/IL-6R axis, as well as JAK-STAT signaling inhibitors (*i.e.* bariticitinib, ruxolitinib, fedtratinib) have raised expectations, prompting pilot studies and clinical trials (44, 45). By showing elevated IFN-inducible chemokines as well as IL-6 at the end of the first week of disease in patients developing severe *versus* milder Covid-19, our findings are informative on the rationale to the therapeutic exploitation of JAK/STAT inhibitors or for cytokine targeting antibodies in patients who develop severe Covid-19. Because JAK/STAT activation is triggered by a wide range of cytokines, our data support its broader inhibition as valid therapeutic candidate to finely modulate the pro-inflammatory cascade downstream of single cytokine signaling, possibly redirecting the disastrous inflammatory response toward disease containment.

Likewise, the most thorough understanding adaptive immunity fingerprints of protective immunity *versus* immune-mediated enhancement of SARS-CoV-2 pathology will be essential to the evaluation and design of a vaccine. By demonstrating similar virus-specific T-cell response, our findings comfort on the presence of M, S and N T-cell response as correlate of acute Covid-19 irrespective of disease severity that will need to be further profiled in the course of disease and convalescence to further inform the requisites of candidate Covid-19 vaccine (16, 20).

## Data Availability Statement

The raw data supporting the conclusions of this article will be made available by the authors, without undue reservation.

## Ethics Statement

The studies involving human participants were reviewed and approved by ASST Santi Paolo and Carlo ethics committee. Patients/participants provided their written informed consent to participate in this study.

## Author Contributions

CT designed the study, analyzed, and interpreted the data, designed the figures, and wrote the manuscript. EC designed the study, performed experiments, analyzed, and interpreted the data designed the figures, and wrote the manuscript. MG performed the experiments. RB interpreted the data and wrote the manuscript. Ad’A helped with interpreting the results and edited the manuscript. GM conceived and designed the study, interpreted the data and wrote the manuscript. All authors contributed to the article and approved the submitted version.

## Funding

This work was supported by grants from Fondazione Cariplo in collaboration with Regione Lombardia and Fondazione Umberto Veronesi (Rif. 2020-1355 and 2020-1376). This work has been presented in part at the *COVID-19 Virtual Conference* (https://covid19.aids2020.org/9); oral poster “C-AIDS2020-10970”), 10-11 July 2020.

## Conflict of Interest

The authors declare that the research was conducted in the absence of any commercial or financial relationships that could be construed as a potential conflict of interest.
