# Supplementary material for: Heightened Circulating Interferon-Inducible Chemokines, and Activated Pro-Cytolytic Th1-Cell Phenotype Features Covid-19 Aggravation in the Second Week of Illness
Source: Front Immunol. 2020 Oct 20;11:580987. doi: 10.3389/fimmu.2020.580987 (PMC7606391; doi:10.3389/fimmu.2020.580987)
Supplement: Supplementary file 1 [file DataSheet_1.pdf]

frontiers

Supplementary Material

Heightened Circulating Interferon-Inducible Chemokines, and Activated Pro-Cytolytic Th1-Cell Phenotype Features Covid-19 Aggravation in the Second Week of Illness

Camilla Tincati[1§], E. Stefania Cannizzo[1§], Mauro Giacomelli[2], Raffaele Badolato[2], Antonella d'Arminio Monforte[1], and Giulia Marchetti[1]*

[1]Department of Health Sciences, Clinic of Infectious Diseases, ASST Santi Paolo e Carlo, University of Milan, Italy; [2] Dipartimento di Scienze Cliniche e Sperimentali, Università degli Studi di Brescia, ASST Spedali Civili and A. Nocivelli Institute of Molecular Medicine, c/o Spedali Civili, 25123 Brescia, Italy

[§] Camilla Tincati and E. Stefania Cannizzo equally contributed to the manuscript

* Corresponding author: Giulia Marchetti, MD, PhD, Clinic of Infectious Diseases - Department of Health Sciences - University of Milan, ASST Santi Paolo e Carlo, Via A Di Rudinì 8 - 20142 – Milan Italy
Phone: +39 02 81843064 - Fax: +39 02 81843054 - e-mail: giulia.marchetti@unimi.it

Running title: Chemokines, T-response in severe Covid19
.
Keywords: Covid-19, SARS-CoV-2, Immunity, S/M/N protein-reactive T-cells, immunopathology

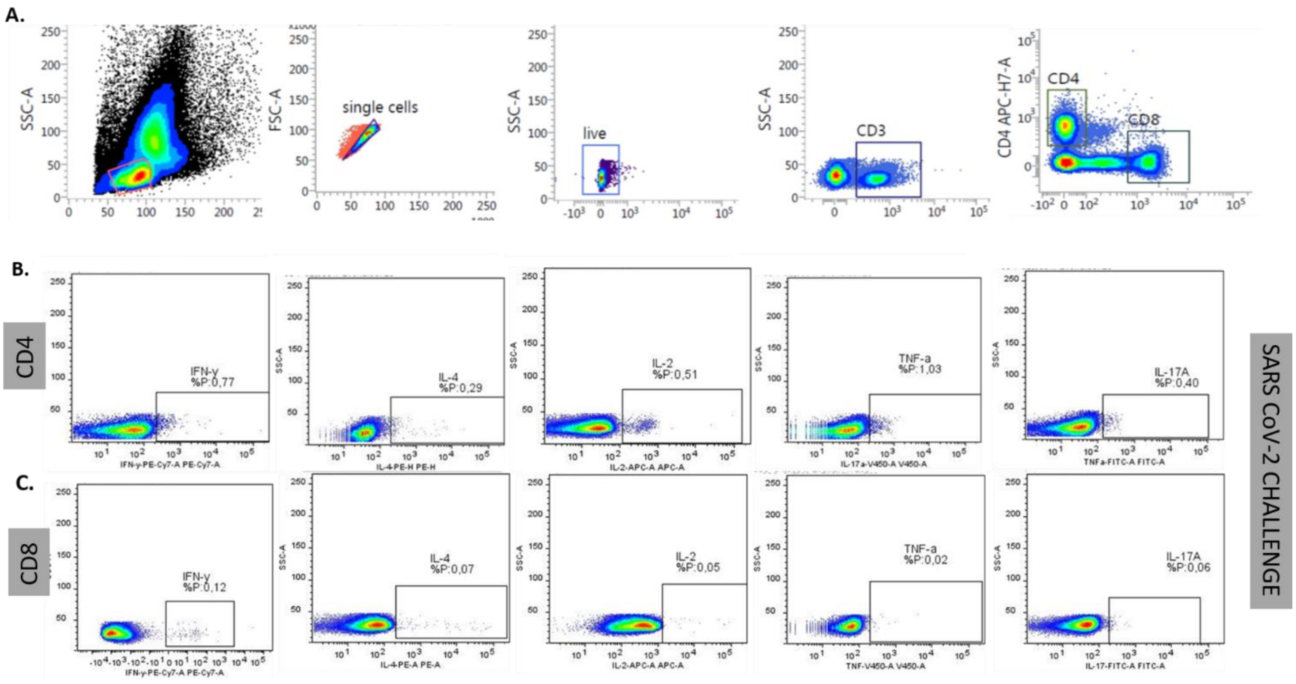

Supplementary figure 1: Gating-strategy for identification of SARS-CoV-2-reactive CD4+ and CD8+ T cells .

Peripheral blood mononuclear cells were stimulated with SARS-CoV-2 pool for 16h. Brefeldin A was added after 1h to block the cytokine secretion. Representative plots illustrate the gating strategy. **A**. Lymphocytes were identified, single cells , live T cells and CD3 were identified and subdivided into CD4+ and CD8+ T cells. These cells were further analyzed for the expression of interleukin (IL) 2, interferon γ (IFNγ), tumor necrosis factor (TNFα), IL4 and IL-17A. **B.** CD4 Antigen-reactive cytokine production was analyzed by the frequency of CD4+ IFN-γ/IL-4/IL-2 /TNFα or IL-17A producers. **C.** CD8 Antigen-reactive cytokine production was analyzed by the frequency of CD8+ IFN-γ/IL-4/IL-2 /TNFα or IL-17A producers.

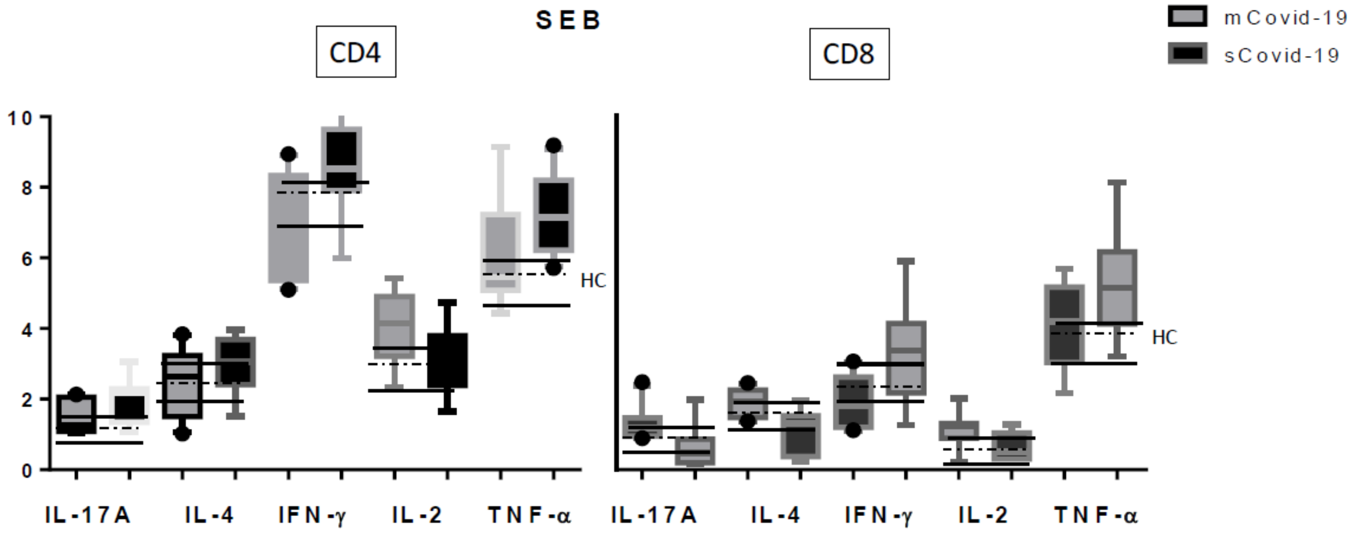

Supplementary figure 2: T cell cytokine secretion upon Staphylococcus aureus enterotoxin B (SEB): Severe Covid-19 (sCovid-19) vs mild Covid-19 (mCovid)

Peripheral blood mononuclear cells (PBMC) were isolated from blood samples of unselected 20 COVID-19 patients (n=10 sCovid-19 and n=10 mCovid-19). 1,5 $*10^6$PBMC were incubated for 16h with Staphylococcus aureus enterotoxin B (SEB) at 1 µg/ml as a positive control and analyzed with flow cytometry. Frequencies of interferon γ (IFNγ)-, tumor necrosis factor α (TNFα)-, interleukin (IL) 2-, IL4-, and IL-17A producing antigen-specific CD4+ and CD8+ T cells upon (A) In each graph, the columns represent the median values, while the error bars indicate the 10th-90th percentile range . Frequencies were corrected by background subtraction as determined in nonstimulated controls. Dotted lines indicate the median levels of healthy controls (HC) from archived material. No major differences in CD4 and CD8 cytokines productions were observed between mCovid-19 and sCovid-19. Comparison between groups (sCovid-19 vs mCovid-19) Mann Whitney test.

Supplementary Table 1. Soluble factors and lymphocyte subset analysis in patients with COVID-19

	Normal range	mCovid-19 n=20	sCovid-19 n=20	p
**CXCL10/IP-10, pg/ml**	52,53-131,9	1806 (728,5-3940)	7069 (2299-21893)	**0,02**
**CCL2/MCP-1, pg/ml**	11,10-22,07	96 (41,25-137,5)	141 (73,25-223,5)	0,22
**CXCL9/MIG, pg/ml**	58,82-165,9	98 (69,25-123)	149 (131-233)	**0,01**
**CCL5/RANTES, ng/ml**	2377-7838	10680 (4424-16645)	9094 (3413-12774)	0,54
**CXCL8/Il-8, pg/ml**	0-0	7,50 (4,75-14,50)	18,50 (7,75-26,25)	**0,06**
**IFN-α, pg/ml**	0-0,89	9,23 (0-49,56)	6,65 (0-30,87)	0,67
**IFN-γ, pg/ml**	0-0	0,63 (0-1,37)	0,83 (0-2,13)	0,7
**IL-1β, pg/ml**	0-0,7	2,29 (0-4,23)	0 (0-1,17)	**0,04**
**IL-4, pg/ml**	0-0	0 (0-0,22)	0 (0-0)	0,64
**IL-5, pg/ml**	0-0,31	0,22 (0,04-0,48)	0,40 (0,28-0,55)	0,25
**IL-6, pg/ml**	0-0,62	17,12 (7,27-32,21)	126,2 (42,37-317,6)	**0,004**
**IL-10, pg/ml**	0-0	5,45 (1,2-6,93)	10,46 (5,7-14,49)	**0,01**
**IL-12, pg/ml**	0-1,82	0,35 (0-6,02)	0 (0-0)	0,07
**IL-17A, pg/ml**	0-0	0 (0-1,54)	0 (0-1,38)	0,75
**TNF-α, pg/ml**	0-0	0 (0-0,91)	0 (0-0)	0,27
**ROS, ng/ml**	1,89-4,5	4,7 (3,1-6,02)	7,9 (5,4-10,14)	**0,003**
**CD3+, %**	48-71	74 (68-77)	71 (69-72)	0,45
**CD3+CD4+, %**	31-60	46 (38-51)	38 (35,50-53)	0,67
**CD3+CD8+, %**	21-36	28 (26-31)	29 (19-34)	1
**B cells, %**	5-10	3,77 (1,79-10,82)	7,10 (0,79-12,79)	0,85
**B cells activated, %**	0,19-0,89	0,29 (0,09-1,08)	0,61 (0,09-0,99)	0,96
**DC, %**	10-19,3	13,35 (7,41-18,84)	11,73 (6,76-18,89)	0,38
**NK, %**	5-21	13,89 (8,58-20,16)	15,87 (10,47-25,85)	0,7
**Th17, %**	3,5-7,7	13,55 (4,29-17,51)	9,54 (4,75-12,70)	0,60
**Treg, %**	1,2-3,8	5,44 (3,35-6,80)	5,84 (4,67-7,86)	0,52
**TFH, %**	4,7-16,30	8,06 (3,31-14,85)	6,08 (1,79-9,02)	0,9
**CD4+CM, %**	11-20,85	23,86 (17,22-31,41)	21,82 (8,79-28,69)	0,36
**CD4+naïve, %**	10,18-36,40	22,49 (14,16-30,62)	37,24 (17,95-57,97)	0,10
**CD4+TD, %**	7,1-8,9	4,26 (2,53-5,98)	4,65 (2,93-17,05)	0,52
**CD4+EM, %**	44,8-60,3	46,03 (41,69-54,56)	26,52 (17,20-44,85)	**0,03**
**CD8+CM, %**	0,07-0,2	0,28 (0,10-0,70)	0,26 (0,01-0,49)	0,28
**CD8+naïve, %**	3,9-15,3	14,46 (8,46-30,38)	29,46 (18,63-63,25)	0,19
**CD8+TD, %**	46-71	49,92 (41,65-70,44)	50,03 (27,24-63,86)	0,73
**CD8+EM, %**	29,30-35	25,59 (17,62-35,41)	13,27 (7,77-24,80)	**0,02**
**Classical Monocytes, %**	80-83,3	77,02 (66,35-79,77)	68,23 (48,04-82,30)	0,63
**Intermediate Monocytes, %**	2,5-3,2	2,30 (1,26-4,56)	1,69 (1,03-2,96)	0,43
**Non classical Monocytes, %**	4,7-6,1	9,37 (4,54-13,10)	16,80 (12,95-23,22)	**0,03**
**CD4+activated, %**	4,3-7,6	9,51 (8,13-16,22)	17,58 (10,62-25,52)	**0,06**
**CD4+GrB+, %**	1,5-4,08	8,50 (3,33-22,23)	16,30 (9,53-29,52)	**<0,0001**
**CD4+PrF+, %**	35,20-47,1	41,88 (27,55-62,68)	67,50 (34,43-78,55)	0,22
**CD4+GrB+PrF+, %**	9,2-23,5	9,49 (8,13-16,22)	17,58 (10,61-25,52)	**0,06**
**CD8+activated, %**	20-61	33,13 (18,92-52,63)	38,42 (32,98-76,02)	0,18

**CD8+GrB+, %**	10,2-18,9	41,91 (26,75-62,49)	67 (33,90-78,51)	0,27
**CD8+PrF+, %**	5,5-22	9,60 (6,19-23,98)	22,95 (11,70-32,40)	**0,05**
**CD8+GrB+PrF+, %**	31,8-48,3	36,84 (33,93-45,88)	45,28 (36,89-77,50)	0,09

∗Data are presented as median (Interquartile range; IQR), statistical analyses: Mann Whitney test. p-values among 2 groups (**mCovid-19** : mild Covid-19 patients and **sCovid-19**: severe Covid-19 patients).**CXCL-10**: C-X-C Motif Chemokine Ligand 10; **IP-10**: Interferon gamma-induced protein 10 ; **CCL2:** C-C Motif Chemokine Ligand 2; **MCP-1**: Monocyte Chemoattractant Protein 1; **CXCL9:** C-X-C Motif Chemokine Ligand 9;**MIG**: monokine induced by gamma interferon; **CCL5:** C-C Motif Chemokine Ligand 5; **RANTES:** Regulated upon Activation, Normal T Cell Expressed and Presumably Secreted; **CXCL8:** C-X-C Motif Chemokine Ligand 8; **IFN:** Interferon; **IL:** Interleukin; **TNF α**: Tumor Necrosis Factor α; **ROS**: reactive oxygen species; **CD:** cluster of differentiation**; B cells activated** (CD19+CD80+) **DC:** dendritic cells; **NK:** natural killer cells; **Th17:** T Helper 17 cells; **Treg**: Regulatory T cells; **TFH**: T Follicular Helper cells; **CM:** Central Memory; **TD:** Terminally Differentiated; **EM:** Effector Memory; **GrB**: Granzyme B; **PrF:** perfotin
